# Flexural Strength, Monomer Release, and Wear of Occlusal Splint Materials Fabricated Through Conventional, Milled, or 3D‐Printed Methods

**DOI:** 10.1002/cre2.70361

**Published:** 2026-04-26

**Authors:** Nurul Liyana Aminuddin, Haralampos Petridis

**Affiliations:** ^1^ Department of Prosthodontics UCL Eastman Dental Institute London England UK; ^2^ Department of Restorative Dentistry, Faculty of Dentistry University Malaya Kuala Lumpur Federal Territory Kuala Lumpur Malaysia

**Keywords:** flexural strength, monomer release, occlusal splint, wear resistance

## Abstract

**Objective:**

To evaluate the flexural strength, monomer release, and wear resistance between conventional, milled polymethylmethacrylate (PMMA), and 3D‐printed resins built at 90° and 60° printing angles for occlusal splints.

**Materials and Methods:**

60‐rectangular and 100‐disc specimens were fabricated from heat‐cured PMMA (Oracryl [HP], Bracon Dental, United Kingdom), milled PMMA (Kerox Premia [KP], Kerox Dental, Hungary), and 3D‐printed resins (FreePrint Splint2.0 [FS], Detax, Ettlingen, Germany, and KeySplint Hard [KS], Keystone Industries, Myerstown, USA) at 90° and 60° printing angles. Specimens for flexural strength and wear tests were immersed immediately in 37°C water for 50 h and thermally aged for 20,000 cycles. Flexural strength was evaluated using a three‐point bend test. Wear was tested using a chewing simulator for 140,000 cycles, and volume loss was calculated using Autodesk MeshMixer software. Monomer release was analyzed via UV spectrophotometry over 7 days. Statistical analysis was performed using the Shapiro–Wilk test and one‐way ANOVA with Tukey's multiple comparison tests.

**Results:**

KP showed the highest mean flexural strength (115.5 ± 5.3 MPa, *p* < 0.0001), followed by HP (86.6 ± 10.8 MPa, *p* < 0.0001), with 3D‐printed resin showed the lowest. Meanwhile 90° FS showed greater flexural strength (60.5 ± 3.8 MPa) compared to 60° FS (*p* < 0.001) and KS (*p* < 0.01). The difference between 90° and 60° KS were not statistically significant (*p* > 0.05). Monomer release peaked on Day 3 for all groups, with KS consistently showing the highest concentration (29.7 ± 3.6 ppm), followed by FS (28.8 ± 3.8 ppm), HP (27.9 ± 4.9 ppm), and lastly, KP showed the lowest concentration (24.9 ± 3.8 ppm). KP demonstrated the lowest mean volume loss (2.5 ± 1.3 mm^3^, *p* < 0.01), followed by HP (4.4 ± 1.7 MPa), whereas 3D‐printed resin showed the highest. No significant wear differences were observed between 90° and 60° printing angles.

**Conclusion:**

Milled PMMA outperformed other materials, followed by conventional PMMA, while 3D‐printed resin showed inferior performance in flexural strength, wear resistance, and monomer release. Printing angles significantly influenced flexural strength but not wear properties in 3D‐printed resins.

## 1 Introduction

Occlusal splint is a removable appliance that is worn over the teeth to protect them from damage or to manage symptoms of temporomandibular joint dysfunctions, including parafunctional habits such as bruxism (Klasser and Greene 2009). This device should be mechanically stable to withstand high occlusal forces, chemically stable, and biocompatible to function in the oral cavity regardless of the different materials and techniques used to fabricate the device.

Occlusal devices can be conventionally manufactured using various polymerization activation techniques, such as heat‐cured, self‐cured, or light‐cured acrylic resin, through methods like flask‐pack‐press and injection moulding (Väyrynen et al. 2016). Conventionally processed PMMA is durable with high tensile strength but brittle under impact and has low dimensional stability due to residual monomer release and incomplete polymerization, which eventually led to increased creep and fracture susceptibility (Powers and Wataha 2013).

Currently, the introduction of Computer‐Aided Design and Computer‐Aided Manufacturing (CAD/CAM) technology has revolutionized the splint fabrication by replacing traditional analog technique with digital workflows (Alghazzawi 2016). There are two methods for digital fabrication, which are subtractive manufacturing, which involves milling, and additive manufacturing, which is known as 3D printing (Alghazzawi 2016). The milling process helps to optimize and simplify the conventional occlusal splint manufacturing process in terms of precision, trueness, durability, and increased quantity of production in a shorter time. Milled PMMA has high flexural strength, is highly precise, has good thermal stability, excellent dimensional accuracy, and biocompatible, making it suitable for applications that require a material with high load‐bearing properties (Angelara et al. 2023). When compared with subtractive manufacturing, 3D printing is more cost‐efficient when small volumes or small numbers are needed. The disadvantages of additive manufacturing are the polymerization shrinkage and the post‐polymerization process, which adds to the cost and time (Abduo et al. 2014). Furthermore, the finishing of the surface texture could affect the dimensional accuracy of the objects (Abduo et al. 2014).

Flexural strength is an important property that reflects the ability of the material to withstand high occlusal forces. This property is clinically relevant in preventing the splint from being damaged and abraded during mastication and cleaning (Maleki et al. 2024).

Biocompatibility of acrylic resins has been debated among researchers, concluding that acrylic resin exposure may result in local irritation, ulceration, and burning mouth sensations (Gautam et al. 2012). During the polymerization process, the incomplete conversion from monomers is converted into stable polymers via an addition reaction, resulting in the release of residual monomers and other chemical products such as methyl methacrylate, formaldehyde, methacrylic acid, benzoic acid, or dibutyl phthalate (Singh et al. 2013; Wedekind et al. 2021). This indicates that the biocompatibility of resins is influenced mainly by the chemical composition of the material as compared to the manufacturing method (Bürgers et al. 2022).

Wear is a process that involves continuous loss and deformation of material from a surface due to mechanical motion between the two surfaces. The chemical composition of the material influences the wear rate and longevity of the occlusal splint. Meanwhile, the wear resistance of 3D‐printed resin may be affected by the angle and position of the manufacturing, with one study showing that the 0° position produced greater wear resistance as compared to the 90° position during the printing process (Grymak et al. 2022a).

With the advancement of digital dental technologies, there are new materials and techniques to produce occlusal splints in a more time‐efficient manner compared to conventional techniques. The evidence related to the properties, biocompatibility, and build angle of the 3D‐printed resin is scarce, as the technology is still evolving as compared to the conventional method and materials.

The aim of this in vitro study was to evaluate the flexural strength, monomer release, and wear resistance between conventional PMMA, milled PMMA, and two different 3D‐printed resins built in different printing angles (90° and 60°) for occlusal splint fabrication. The null hypothesis was that there would be no statistically significant differences in flexural strength, monomer release, or wear properties between conventional PMMA, milled PMMA, and 3D‐printed resins built in different printing angles (90° and 60°).

## 2 Materials and Methods

The comprehensive details regarding the manufacturer and material composition of conventional heat‐cured PMMA, milled, and 3D‐printed resins were presented in Table 1.

**Table 1:** Type of manufacturing method, code, product name, manufacturer, and composition of the tested materials.

Fabrication method	Code	Product name	Manufacturer	Composition	Property evaluated
Conventional PMMA	HP	Oracryl	Bracon Dental, East Sussex, United Kingdom	Polymethylmethacrylate (PMMA)	Flexural strength, monomer release, and wear test
Milled PMMA	KP	Kerox Permia	Kerox Dental, Soskut, Hungary	Polymethylmethacrylate (PMMA)	
3D‐printed	FS	FreePrint Splint 2.0	Detax GmbH, Ettlingen, Germany	Isopropylidenediphenol peg‐2 dimethacrylate 90% <95%	
	KS	KeySplint Hard	Keystone Industries, Myerstown, USA	Methyl methacrylate (MMA), photo‐initiator, inhibitor, pigment	

### 2.1 Sample Preparation

Bar specimens (65 × 10 × 3.3 mm, *n* = 10/group) were fabricated for flexural strength test according to ISO 20795‐1 (2013). Disc specimens (10 × 3 mm, *n* = 10/group) were fabricated for monomer release and wear tests. The bars were divided into six groups for the flexural strength test. The discs were divided into four groups and six groups to test for the monomer release and wear resistance, respectively. The sample size was determined based on findings from a pilot study.

### 2.2 Conventional Manufactured Specimens

**Figure 1:**
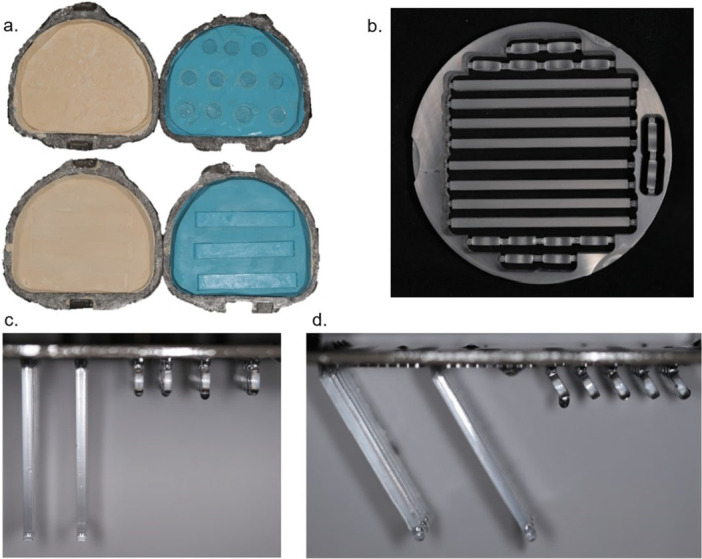
Fabrication of bars and disc specimens (a) conventional PMMA, (b) milled PMMA, (c) 90° 3D‐printed, and (d) 60° 3D‐printed.

The heat‐cured PMMA samples were prepared using the compression moulding technique. Bar and disc templates were designed with DesignSpark Mechanical software (Ansys Inc.) and milled from wax blocks using milling unit M1 heavy dry machine (Zirkonzahn). Condensation‐cured silicone lab putty (Kulzer) was placed in the lower half of the flask, and templates were embedded in the lab putty. The upper half of the flask was filled with a 50/50 mixture of Type III dental stone and Plaster of Paris, as shown in Figure 1a. After the gypsum was set, the flask was opened, and the templates were removed. According to the manufacturer's instructions, the heat‐cured acrylic resin was mixed, poured into the mould, pressed at 2 bar for 5 min, and cured in a water bath at 73.5°C for 7 h before cooling and deflasking.

### 2.3 Digital Manufactured Specimens

For digitally processed methods, the specimens were virtually designed with Designspark Mechanical software (Ansys Inc.) according to the dimension for the flexural strength test, chemical test, and wear test, respectively. The design was exported as STL file and imported into a nesting software (Zirkonzhan). For the subtractive manufacturing group, the specimens were milled from prefabricated PMMA disc (KP) using milling unit M1 heavy wet machine (Zirkonzahn), as shown in Figure 1b.

For the additive manufacturing group, the specimens were designed in Netfabb software (Autodesk) at two different build angles, which were perpendicular and 60° angle to the build platform with supporting structures. The specimens were printed using the 3D printing machine Straumann P Series Rapidshape P30+ (Straumann), as shown in Figure 1c,d. According to ISO 20795‐1 (2013), all specimens were polished under wet condition using P1200 grit silicon carbide paper (Paper SiC P1200, Struers GmbH) on a BETA Twin Variable Speed Grinder Polisher (Buehler) operated at 150 rpm (ISO 20795‐1 2013).

### 2.4 Aging Regime

**Figure 2:**
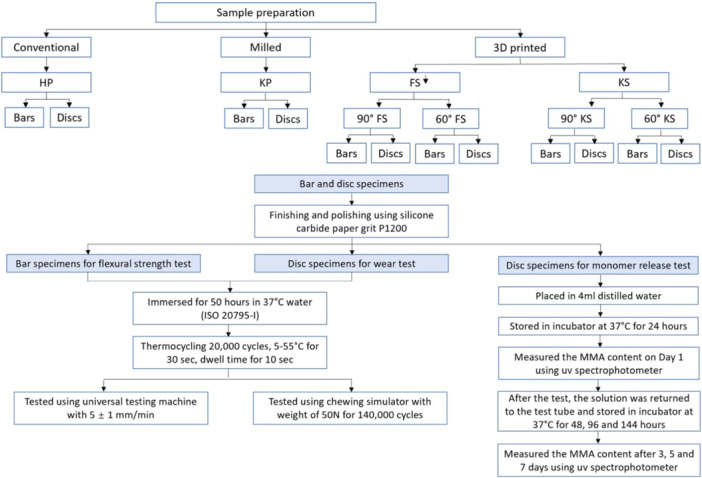
Overall diagram of the sample preparation and testing.

After polishing, the specimens for flexural strength and wear tests were stored in 37°C water for 50 h, following the (ISO 20795‐1 2013) guidelines. Then, the specimens were subjected to thermal aging using a Thermocycler 1100 (SD Mechatronik). The specimens were placed in a basket and cycled for 20,000 cycles between the two chambers with each cycle involving a 30‐second immersion in 5°/55°C, followed by a 10‐second dwell time. The overall diagram of the sample preparation and testing of the samples is shown in Figure 2.

### 2.5 Flexural Strength Test

The bar specimens were positioned symmetrically on the base of a universal testing machine (AGS‐X Series, Shimadzu). The load force was gradually increased at a consistent rate of 5 ± 1 mm/min until the specimen fracture. The flexural strength of each specimen was calculated using the following formula:

$$\sigma =3\text{Fd}/2{\text{wh}}^{2},$$

*F* is the maximum load, in newtons, exerted on the specimen;

*d* is the distance, in millimeters, between the supports;

*w* is the width, in millimeters, at the center of the specimen measured immediately prior to testing; and

*h* is the height, in millimeters, at the center of the specimen measured immediately prior to testing.

### 2.6 Chemical Testing: Monomer Release

The bar specimen was placed in a sterilized tube with 4 mL of distilled water and stored in an incubator (Hera Cell 150, Heraeus Kulzer) at 37°C for artificial aging.

#### 2.6.1 Absorption Maximum of Pure MMA Solution

A spectrophotometer (UNICAM UV500, Thermo Spectronic) and quartz cells (UV–Vis Spectroscopy cells, Perkin Elmer Lab Solutions) were utilized to measure the absorption of residual MMA eluted from the specimen at a wavelength spectrum of 220 nm (Keul et al. 2020).

#### 2.6.2 Calibration Curve

A standard solution was prepared by mixing 10 μL of pure MMA (Scientific Laboratory Supplies, Nottingham, United Kingdom) with 9990 μL of distilled water (1000 ppm of MMA) (Keul et al. 2020). This solution was stored at 37°C in an incubator Heracell 150 (Thermo Electron GmbH) for 12 h to enhance molecular interaction. From this standard solution, five further solutions were prepared with concentrations of 10, 20, 30, 40, and 50 ppm by diluting the standard solution with distilled water (Keul et al. 2020). Each solution was measured using a spectrophotometer, and the absorbance values were recorded. The slope (*m*) of the calibration curve was then determined, allowing the MMA concentration in the specimens' elutions to be calculated using the following formula:

$$A=1/4\;m*C_{\text{MMA}}.$$

#### 2.6.3 Determination of the MMA Content in the Storage Solution

Each specimen solution was measured at a wavelength of 220 nm using UV spectrophotometry to determine the monomer content after 24 h. Regarding the 3D‐printed groups, the 90° printing angle specimens were chosen for testing since differences in printing angles did not influence the results of the monomer release test. Absorbance values were recorded in the UV WinLab Software (Perkin Elmer, Perkin Elmer Inc.) (Keul et al. 2020). The solution was replaced with 4 mL of distilled water every 24 h and stored in the incubator until Day 7. New readings of MMA elution content were measured after 3, 5, and 7 days using a UV spectrophotometer.

### 2.7 Wear Test

The wear test was performed using a chewing simulator (SD Mechatronik CS‐4.4). The sample resin was placed in the lower holder. A Steatite ball, 6 mm in diameter, was embedded in autopolymerizing acrylic resin within a plastic ring and fixed in the upper antagonist using a fastening screw. The chewing simulator operated at 1.5 Hz with a 5 kg vertical load, generating a chewing force of 50 N for 140,000 cycles (Lutz et al. 2019). The wear patterns were scanned using an S600 ARTI scanner (D1000 desktop scanner, Zirkonzahn). The STL file was analyzed using the CAD MeshMixer software (Autodesk MeshMixer Vol 3.5.474; Autodesk Inc.) to calculate the volume loss by subtracting the original volume from the volume obtained (Lee et al. 2022).

### 2.8 Statistical Analysis

The raw data were collected in a spreadsheet using Microsoft Excel and GraphPad Prism (GraphPad Software Inc.), and IBM SPSS Statistics version 28.0.1 (IBMSPSS Inc.) software was used to conduct the statistical analysis for each test. The flexural strength, monomer release, and total wear loss were analyzed by using the Shapiro–Wilk test and one‐way ANOVA with Tukey's multiple comparison tests to identify differences between materials.

## 3 Results

### 3.1 Flexural Strength Test

**Figure 3:**
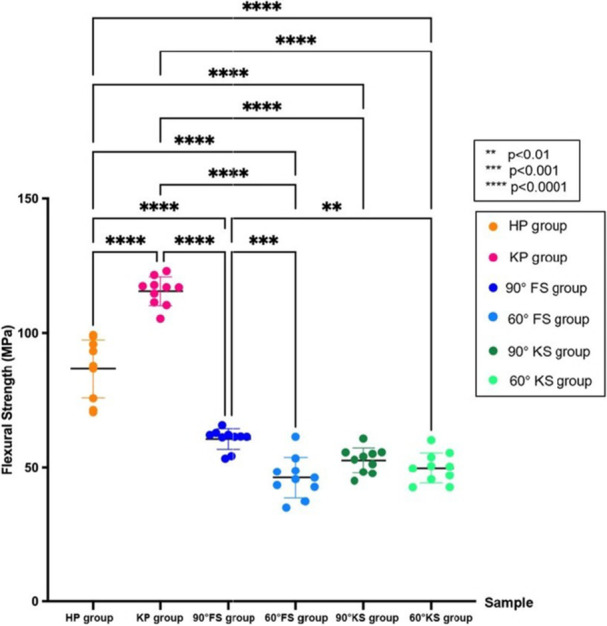
Dot plots show the mean value of flexural strength (MPa) with standard deviation (SD), where * indicates statistical differences.

The KP group showed the highest mean flexural strength values (115.5 ± 5.3 MPa) (*p* < 0.0001), outperforming all other groups (Table 2 and Figure 3). The HP group (86.6 ± 10.8 MPa) had statistically significantly higher flexural strength than the FS and KS groups (*p* < 0.0001).

**Table 2:** Descriptive data of flexural strength (MPa) by group

Group	Mean	SD	Min	Max	95% CI for mean	
					Min	Max
HP	86.6	10.8	70.4	99.3	78.9	94.3
KP	115.5	5.3	105.3	122.9	111.7	119.3
90° FS	60.5	3.8	53.3	65.6	57.8	63.2
60° FS	46.1	7.6	34.9	61.3	40.7	51.5
90° KS	52.6	4.7	44.8	60.7	49.2	56.0
60° KS	49.7	5.7	42.4	60.1	45.6	53.8

Abbreviations: FS, 3D‐printed FreePrint Splint 2.0; HP, heat polymerized PMMA; KP, milled Kerox Premia PMMA; KS, 3D‐printed KeySplint Hard.

Comparing the different printing angles of the 3D‐printed resins, the 90° FS group had a statistically significant higher mean flexural strength (60.5 ± 3.8 MPa) compared to both 60° FS (*p* < 0.001) and 60° KS groups (*p* < 0.01). However, the differences between 90° KS and 30° KS or 30° FS were not statistically significant.

### 3.2 Monomer Release Test

**Figure 4:**
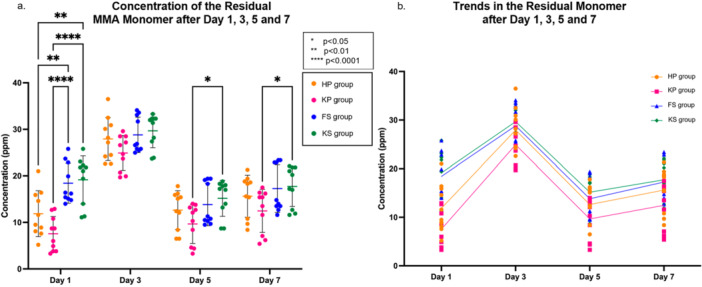
Dot plots show the (a) mean residual monomer concentration (ppm) over 7 days with SD, where * indicates statistical differences and (b) trends in residual monomer concentration (ppm) after Days 1, 3, 5, and 7.

The KS group had the highest concentration at 19.2 ± 5.2 ppm on Day 1, which was statistically different when compared to both the KP group (*p* < 0.0001) and the HP group (*p* < 0.01) (Table 3 and Figure 4a). The KP group exhibited the lowest concentration of residual monomer at 7.6 ± 3.7 ppm, which was statistically significantly lower than the concentrations observed in the FS and KS groups (*p* < 0.0001). By Day 3, the concentrations became more clustered around 24.9 to 29.7 ppm, with no significant differences. Days 5 and 7 show similar clustering with concentrations ranging from 9.7 to 15.2 ppm and 12.5 to 17.7 ppm, respectively. Overall, the concentration of residual monomer fluctuated overtime during the 7‐day incubation period (Figure 4b). From Day 1 to Day 3, there was a significant increase in residual monomer concentration in all groups. By Day 3, all groups exhibited a peak in concentration, with the KS group reaching the highest concentration of monomer release at 29.7 ± 3.6 ppm. By Day 5, the concentrations dropped significantly for all groups. By Day 7, the concentrations increased again for all the groups but did not reach the peak levels observed on Day 3. The KS group remained the highest concentration of monomer, while the KP group had the lowest concentration until Day 7. These differences were statistically significant between the KP and KS groups on Days 5 and 7 (*p* < 0.05).

**Table 3:** Descriptive statistics of MMA elution in ppm (Mean, SD: Standard deviation).

Group	MMA elution (ppm)		Day 1	Day 3	Day 5	Day 7
**HP**	Mean		11.9	27.9	12.6	15.6
	SD		4.9	4.6	4.2	4.5
	Min		5.2	22.6	6.5	8.4
	Max		21	36.5	17.8	21.3
	95% CI for Mean	Min	10.2	26.3	11.1	14.0
		Max	13.7	29.6	14.1	17.2
**KP**	Mean		7.6	24.9	9.7	12.5
	SD		3.7	3.8	4.2	4.6
	Min		3.3	19.7	3.3	5.4
	Max		12.9	29.6	13.8	16.4
	95% CI for Mean	Min	6.3	23.5	8.2	10.9
		Max	8.9	26.3	11.2	14.2
**FS**	Mean		18.4	28.8	13.8	17.3
	SD		4.3	3.8	4.5	5.1
	Min		14	25	9.5	11.6
	Max		25.8	34.1	19.4	23.4
	95% CI for Mean	Min	16.9	27.4	12.2	15.5
		Max	19.9	30.2	15.4	19.1
**KS**	Mean		19.2	29.7	15.2	17.7
	SD		5.2	3.6	3.9	4.3
	Min		11.1	23.7	8.7	11.6
	Max		25.8	33.3	18.8	21.8
	95% CI for Mean	Min	17.3	28.4	13.8	16.2
		Max	21.1	31.0	16.6	19.2

Abbreviations: FS, 3D‐printed FreePrint Splint 2.0; HP, heat polymerized PMMA; KP, milled Kerox Premia PMMA; KS, 3D‐printed KeySplint Hard; Min, Minimum; Max, Maximum.

### 3.3 Wear Test

**Figure 5:**
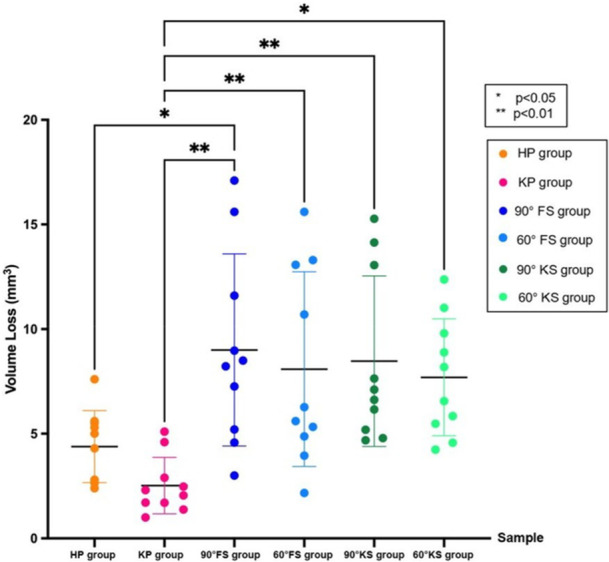
Dot plots show the mean value of volume loss (mm³) with standard deviation (SD), where * indicates statistical differences.

The KP group showed the lowest mean volume loss (2.5 ± 1.3 mm^3^) compared to the other five groups. The difference between the mean volume loss of the KP group and the 3D‐printed groups was statistically significant (*p* < 0.01) but the difference was not significant when compared to the HP group (Table 4 and Figure 5). No statistically significant difference was noted when comparing the mean volume loss among the 3D printed groups, regardless of the built angle.

**Table 4:** Descriptive data of volume loss (mm^3^) by group.

Group	Mean	SD	Min	Max	95% CI for mean	
					Min	Max
HP	4.4	1.7	2.4	7.6	3.2	5.6
KP	2.5	1.3	1.0	5.1	1.6	3.4
90° FS	9.0	4.6	4.6	17.1	5.7	12.3
60° FS	8.1	4.7	3.9	15.6	4.7	11.5
90° KS	8.5	4.1	4.7	15.3	5.6	11.4
60° KS	7.7	2.8	4.2	12.4	5.7	9.7

Abbreviations: FS, 3D‐printed FreePrint Splint 2.0; HP, heat polymerized PMMA; KP, milled Kerox Premia PMMA; KS, 3D‐printed KeySplint Hard.

## 4 Discussion

The aim of this study was to evaluate the flexural strength, monomer release, and wear resistance between conventional PMMA, milled PMMA, and two 3D‐printed resins used for occlusal splint fabrication built in different printing angles (90° and 60°). All tests indicated that conventional and milled PMMA exhibited lower mean values for monomer release and higher mean values for flexural strength and wear resistance compared to the 3D‐printed resin materials. Consequently, the three null hypotheses were rejected.

The 3D‐printed resins exhibited the lowest mean flexural strength with values for the FS and KS groups failing to meet the ISO 20795‐1:2013 standard of 65 MPa (ISO 2013). Flexural strength ranged from 46.1 to 60.5 MPa, aligning with previous studies from Maleki et al. (2024) and Berli et al. (2020) showing similar results for most 3D printed resin materials (ranging from 3.0 to 36.6 MPa after thermocycling) except for the V‐Print Splint (80.6 ± 9.5 MPa). The differences in flexural strength among these 3D‐printed materials may be influenced by their chemical composition, including monomer structure, cross‐linked polymer chains and inorganic fillers, which contribute to the overall strength of the resin (Alp et al. 2019). The curing process, including both the initial layer‐by‐layer polymerization and subsequent post‐curing influence the final degree of polymerization and mechanical properties (Kim et al. 2020). Although all specimens in this study were post‐cured according to manufacturers' instructions, the degree of conversion was not directly measured. Post‐curing procedures have been shown to significantly increase the degree of conversion and improve mechanical properties such as flexural strength by enhancing cross‐linking density and reducing residual monomers. Previous studies have reported that longer post‐curing times and optimized curing protocols improve flexural strength and other mechanical properties of 3D‐printed resin materials (Kim et al. 2020).

The results of our study also indicated that the conventional manufacturing technique exhibited the highest variability in terms of manufacturing consistency, as evidenced by the largest standard deviation (SD) and the widest minimum‐maximum (Min‐Max) range. In contrast, digital techniques demonstrated more standardized and consistent outcomes. This suggested that digital methods offered improved control over the manufacturing process, leading to more predictable and reliable material properties compared to the conventional approach, which may be more prone to inconsistencies and variations (Conceição et al. 2023). The differences in flexural strength between 3D‐printed splints from the KS and FS groups illustrated how material flexibility and manufacturing processes could impact mechanical properties. The statistically significant difference in flexural strength between 90 and 60° FS suggested that the printing orientation greatly affected the structural integrity of FreeSplint 2.0, likely due to variations in layer bonding and resin properties. Conversely, the non‐significant difference between 90° and 60° KS indicated that KeySplint's material composition and manufacturing process provided more consistent flexural strength, regardless of printing orientation. Flexible materials, like those possibly used in FreeSplint 2.0, typically exhibited lower flexural strength due to their higher elasticity, leading to more pronounced differences based on manufacturing conditions (Prpic et al. 2023).

Investigating the biocompatibility of a material is crucial to ensure the safety of dental devices, especially if the device will be used in the mouth. Previous research (Wedekind et al. 2021) showed that 3D‐printed resins exhibited higher biocompatibility compared to milled or conventional resins due to higher levels of residual monomers (Wedekind et al. 2021). However, Wedekind's research only extended the experiment to Day 3, whereas our study found that elution dropped significantly after Day 5. The fluctuating results obtained from this study closely align with those observed in Keul et al. study, which conducted the experiment over 15 days using a similar methodology and showed that monomer release was a continuous process over the 15 days of incubation (Keul et al. 2020). The pattern of residual monomer release observed in this study may reflect the complex diffusion‐controlled elution kinetics of MMA from PMMA materials. Residual monomer trapped within the PMMA network can initially diffuse out rapidly, resulting in an early peak. As the more readily accessible monomer is released, the concentration in the medium may decrease. However, with continued water penetration into the polymer matrix over time, additional monomer from deeper within the structure can become mobilized and elute later, which may explain the observed increase at Day 7. Such non‐linear release profiles have been described for acrylic denture base materials, where residual monomer continues to leach out over days or weeks as equilibration and diffusion occur within the polymer matrix (Vallittu et al. 1995).

The industrial manufacturing process of PMMA blocks for milling could minimize the total MMA elution. This was confirmed by the results obtained in this research, where milled PMMA exhibited the lowest monomer release compared to other manufacturing processes. Srinivasan et al. also demonstrated that milled resins exhibited comparable biocompatibility to conventional heat‐cured resins (Srinivasan et al. 2018). However, the conventional mixing of powder and liquid resulted in greater monomer release than milled PMMA, which could lead to mucosal irritation and tissue sensitization at certain levels (Jorge et al. 2003). Research also comparing polished and unpolished specimens has demonstrated that polishing can reduce residual monomer elution, which was relevant for this investigation (Wedekind et al. 2021).

This study identified a significant difference in wear resistance among occlusal splint materials, with milled PMMA demonstrating the highest wear resistance, followed by the conventional PMMA, and lastly, the 3D‐printed resins showing the least wear resistance. These results were in agreement with previous studies from Gibreel et al. (2022), Grymak et al. (2022a), and Lutz et al. (2019). These differences can be attributed to production methods in which the conventional and milled PMMA are manufactured to create a densely packed PMMA material, resulting in a homogeneous, porosity‐free structure (Arcas et al. 2023). In contrast, 3D‐printed materials were manufactured using photopolymerization‐based additive manufacturing technologies, in which the material is built layer by layer. The printing angle influences the orientation of the layers as well as the position of the supporting structures. An important consideration in evaluating wear resistance was to replicate the materials' experience in the oral environment. To simulate in vivo wear for 12 months, this formula was applied by following the ISO standard 14569‐3, 8.1 bruxing cycles per hour multiplied by 24 h, 365 days, and 2 years, resulting in a total of 141,523 cycles (Grymak et al. 2022a). Thus, the 140,000 cycles were carried out to represent 2 years of contact Grymak et al. 2022b).

In a recent study (Lawson et al. 2024), rigid 3D‐printed materials demonstrated comparable or even greater wear resistance than light‐polymerized, heat‐polymerized, and milled materials; however, the flexible 3D‐printed resins showed relatively lower wear resistance (Lawson et al. 2024). The 3D‐printed specimens were designed at 0° orientation to the platform, which may have influenced these results (Lawson et al. 2024). A 0° printing angle means that the occlusal and fit surfaces are parallel to the build platform, leading to better interlayer adhesion and uniform stress distribution. However, the support structures required for the 0° printing angle can affect the occlusal or fitting surfaces, potentially leading to inaccuracies in the occlusal contact points or fit. Therefore, some angulation is necessary to avoid such errors while providing printing support at the possible expense of lower mechanical properties. The results of our study were consistent with the findings of Grymak et al. (2022a). The 3D‐printed materials demonstrated a trend of increased volumetric loss, attributed to the lattice layering and porous structures inherent in the printing process. Based on the findings, 3D printing materials used for occlusal splint fabrication should be recommended only for short‐term use in everyday clinical practice.

One limitation of our study was the fact that the wear test was conducted under dry conditions which do not simulate the wet conditions of the oral environment and the presence of saliva which may play a significant role in the wear process of dental materials (Yilmaz and Sadeler 2021). Some studies suggested that dry conditions can also be used for specific evaluations, but they may not fully reflect the actual wear behavior seen intraorally and potentially leading to misleading conclusions about the material's performance (Yildiz Domanic et al. 2020). Another limitation of our study was the use of UV spectrophotometry instead of gas chromatography (GC) for the monomer release test, due to the unavailability of GC in the lab. This constraint potentially impacts the precision and specificity of the monomer release measurements. GC is often considered superior to UV spectrophotometry for testing monomer release due to its higher sensitivity, specificity, and ability to separate complex mixtures (Keul et al. 2020). Additionally, GC is a standard method recommended by ISO 20795‐1 for determining the MMA concentrations in liquid (ISO 20795‐1 2013). GC can accurately quantify low levels of residual monomers and provide detailed molecular information, which UV spectrophotometry may not achieve due to interference from other absorbing substances in the sample. Within the limitations of our study and for the specific materials used, milled PMMA would be recommended to be used for long‐term application, such as a post‐operative hard occlusal splint due to its high flexural strength, high wear resistance, and good biocompatibility.

To improve the long‐term clinical use and wear resistance of 3D‐printed splint materials, future research should focus on optimizing these materials with a specific combination of cleaning and post‐polymerization processes. Factors such as printing layer thickness, water storage, and post‐curing can significantly impact the mechanical, physical, and wear properties of 3D‐printed occlusal splints (Perea‐Lowery et al. 2021). Based on the results of our study, 3D‐printed occlusal splints built at a 60° printing angle would be recommended for short‐term clinical applications, typically a few months, due to their lower cost and rapid fabrication process, though their mechanical properties are inferior compared to milled PMMA.

Further research should focus on optimizing the combination of materials, compositions, and printing angles to improve both flexural strength and wear resistance. As this study was conducted in‐vitro, further clinical studies are required to validate these findings and to evaluate the long‐term performance of occlusal splints fabricated from different materials.

## 5 Conclusions

Within the limitations of this study, the following conclusions can be drawn: Milled PMMA outperformed other materials, followed by conventional PMMA, while 3D‐printed resin showed inferior performance in flexural strength and wear resistance. The 90° 3D‐printed groups showed a significantly higher flexural strength compared to the 60° 3D‐printed groups, emphasizing the influence of printing angles on the mechanical properties of 3D‐printed resins. However, the difference in volume loss between the 60° and 90° printing angles was not statistically significant in terms of wear properties. Milled PMMA group exhibited the lowest concentration of residual monomer, followed by conventional PMMA. 3D‐printed groups showed the highest concentration until Day 7. The observed fluctuating results indicated that monomer release was a continuous process over the 7 days of incubation.

## Author Contributions

**Nurul Liyana Aminiddin:** conceptualization, methodology, investigation, formal analysis, data curation, writing – original draft, writing – review and editing. **Haralampos Petridis:** conceptualization, methodology, writing – review and editing, supervision.

## Funding

The authors have nothing to report.

## Ethics Statement

No Ethical approval was required for this in vitro study.

## Conflicts of Interest

The authors declare no conflicts of interest.

## Data Availability

Data available upon request.
